# Pest and Disease Management: Why We Shouldn't Go against the Grain

**DOI:** 10.1371/journal.pone.0075892

**Published:** 2013-09-30

**Authors:** Peter Skelsey, Kimberly A. With, Karen A. Garrett

**Affiliations:** 1 Information and Computational Sciences, James Hutton Institute, Dundee, Scotland, United Kingdom; 2 Division of Biology, Kansas State University, Manhattan, Kansas, United States of America; 3 Department of Plant Pathology, Kansas State University, Manhattan, Kansas, United States of America; Universidade Federal do Rio de Janeiro, Brazil

## Abstract

Given the wide range of scales and mechanisms by which pest or disease agents disperse, it is unclear whether there might exist a general relationship between scale of host heterogeneity and spatial spread that could be exploited by available management options. In this model-based study, we investigate the interaction between host distributions and the spread of pests and diseases using an array of models that encompass the dispersal and spread of a diverse range of economically important species: a major insect pest of coniferous forests in western North America, the mountain pine beetle (*Dendroctonus ponderosae*); the bacterium *Pseudomonas syringae*, one of the most-widespread and best-studied bacterial plant pathogens; the mosquito *Culex erraticus*, an important vector for many human and animal pathogens, including West Nile Virus; and the oomycete *Phytophthora infestans*, the causal agent of potato late blight. Our model results reveal an interesting general phenomenon: a unimodal (‘humpbacked’) relationship in the magnitude of infestation (an index of dispersal or population spread) with increasing grain size (i.e., the finest scale of patchiness) in the host distribution. Pest and disease management strategies targeting different aspects of host pattern (e.g., abundance, aggregation, isolation, quality) modified the shape of this relationship, but not the general unimodal form. This is a previously unreported effect that provides insight into the spatial scale at which management interventions are most likely to be successful, which, notably, do not always match the scale corresponding to maximum infestation. Our findings could provide a new basis for explaining historical outbreak events, and have implications for biosecurity and public health preparedness.

## Introduction

A fundamental question in invasive species and disease ecology concerns the role of spatial heterogeneity and scale in influencing the spatial spread of pests and pathogens or their vectors [1]–[3]. Many pest and pathogen species are distributed and operate across a wide range of spatial scales, and exhibit a great diversity of dispersal mechanisms, from passive transport (involving wind–, splash–, ballistic–, tumble–, gravity–, and water–borne dispersal) to the various forms of animal locomotion (i.e., active transport, involving swimming, walking, gliding and flight). This has thus thwarted the development of a general predictive framework as to how different aspects of scale might influence the interaction of spatial heterogeneity and pest or disease spread.

Recent ecological theory – the dispersal scaling hypothesis (DSH) – posits a unimodal (‘humpbacked’) relationship between the magnitude of dispersal and the grain size (finest scale of patchiness) of a habitat distribution [4]. Under the DSH, the magnitude of dispersal (the number of individuals moving from one patch to another) is predicted to increase with increasing grain size (scale) up to a certain point (the ‘hump’), after which the magnitude of dispersal declines. Initially, dispersal increases with patch size, as larger populations produce more dispersers and larger patches are more attractive or make bigger targets for dispersers. Eventually, however, a maximum scale of patchiness is reached at which there are no further gains in dispersal, and the magnitude of dispersal instead begins to decline with further increases in grain size. This is because increasing the grain size of the landscape also increases the size of the gaps—the non-habitat areas—between patches, which has an overall depressive effect on dispersal. Thus, to summarize, resource patchiness can vary over a spectrum of scales (grain sizes), dispersal processes have a characteristic scale (the dispersal range of the species), and the interplay between these different scales results in a unimodal distribution of dispersal magnitudes. The exact grain size at which dispersal is maximized, however, is expected to depend on other aspects of spatial heterogeneity, such as the amount, quality, and distribution of habitat (or hosts) on the landscape.

As such, the DSH offers a new theoretical framework and quantitative approach for identifying the critical scales at which the scaling of species and their environment coincide, which may then have important implications for assessing the ability and degree to which species might spread throughout the landscape. Not only would this be important in the present context of mapping the potential for pest and disease spread, but it might also permit an evaluation of the likely efficacy of certain management interventions that are applied (or could be) at a particular range of scale(s). Ensuring the coincidence between the scale(s) at which management interventions are applied and the scale(s) at which pests or pathogens are likely to be most susceptible is an obvious and desirable goal in disease or pest management. Thus, this sort of analysis could be of predictive value for assessing future suppressive and containment scenarios, which factor prominently in evaluating biosecurity and public-health preparedness.

As originally formulated, the analytical framework of the DSH incorporates a generalized dispersal function within a simple two–patch (donor–recipient) landscape. In this paper, we refine and extend this theoretical foundation to facilitate the linkage of both dispersal and population dynamics that ultimately give rise to spatiotemporal patterns of pest and disease spread. We first do this analytically, and then develop numerical predictions using a spatially-explicit modeling approach (described below) for the dispersal and spread of a diverse range of economically important pests and diseases: (i) the mountain pine beetle, *Dendroctonus ponderosae*, a major forestry pest responsible for the largest insect-caused forest blight ever observed in western North America [5]; (ii) the bacterium *Pseudomonas syringae*, which infects an extraordinarily wide variety of plants and is therefore one of the (if not the) most-important and best-studied bacterial plant pathogens [6]; (iii) the mosquito *Culex erraticus*, an important vector for many human and animal pathogens, including West Nile Virus [7]; and, (iv) the oomycete *Phytophthora infestans*, the causal agent of potato late blight, which is widely regarded as one of the most costly constraints to attaining global food security [8]–[10]. These species encompass a wide range of dispersal modes and scales, which we model using a variety of deterministic and stochastic methods for dispersal. Going beyond the simple two-patch system of the original DSH formulation, we developed a spatially-explicit modeling approach in which we simulated host distributions (landscapes) by varying the grain (i.e., the cell dimensions of a raster landscape) across a wide range of grain (host-patch) sizes to search for interactions between scale and the magnitude of infestation (defined in terms of either dispersal and/or spatiotemporal spread, as appropriate to the pest or pathogen). We also simulated four major landscape-management strategies aimed at reducing infestation, through manipulation of various aspects of the host distribution: (i) host abundance, (ii) host quality, (iii) matrix permeability, and (iv) aggregation of host areas. These scenarios were designed to reveal potential interactions of scale and management efficacy for each type of pest or pathogen.

We find that all of our simulation results are consistent with the predictions of the DSH: there was a unimodal relationship between the magnitude of infestation and the spatial grain of the host distribution, regardless of the spread process. We also show that manipulation of host pattern modifies only the shape of this relationship, and not the general unimodal form. This is a previously unreported effect that provides insight into the spatial grain (scale) at which pests and pathogens respond to host pattern and thus where management interventions are likely to be most effective, which, notably, do not always match the scale corresponding to maximum infestation.

## Methods

### Theoretical framework – influence of spatial grain on spread

The DSH was originally formulated using a parsimonious, discrete–space model of a single generation of dispersal from a square donor patch to a recipient patch of equal dimensions, where the total number of dispersing agents (e.g., individuals, spores) arriving at the recipient patch, *N* (no.), was determined as the product: *N* = donor density (no. m^−2^)×donor area (m^2^)×dispersal probability (no. m^−2^)×recipient area (m^2^). Here, we refine the original theoretical framework by adopting a continuous-space analogue. This is more computationally intensive but more indicative of the actual density of organisms moving to a recipient patch [11], [12]. In the original formulation the appropriate dispersal function was used to calculate the probability of dispersal from the center of the donor patch to the center of the recipient patch (centroid-to-centroid dispersal) and that value was used for the whole of the recipient patch. Here, a continuous probability density function, *k* (m^−2^) is integrated over patch dimensions (area-to-area dispersal) [11]–[15]:
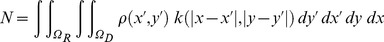
(1)where (*x*,*y*) are locations within the recipient patch, (*x′*,*y′*) are locations within the donor patch, *ρ* (no. m^−2^) is the patch population density, and *k* (|*x−x*′|, |*y*−*y*′|) is the probability of moving from (*x′*,*y′*) to (*x*,*y*) across two-dimensional space. If we set recipient patch and donor patch dimensions to *L* (length), then the distance between donor and recipient patch centers can be written as *βL*, where *β* (−) is a multiplicative factor that adjusts the separation distance between patches: if *β*<1 then the patches overlap, if *β* = 1 then the patches are adjacent, and if *β*>1 then there is a gap between them. In order to quantify the total number of mobile agents dispersing from the donor patch across the whole area of the recipient patch, *Ω_D_* = [−*L*2, *L*/2]×[*βL*−*L*/2, *βL*+*L*/2] and *Ω_R_* = [−*L*/2, *L*/2]×[−*L*/2, *L*/2].

We initially assume an exponential power distribution for our dispersal kernel, as it has the advantage of a flexible shape [16]–[18]. The basic kernel is:
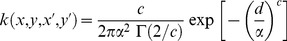
(2)where *c* is a dimensionless shape parameter, *α* is a spread parameter (m), Γ is the gamma function, and 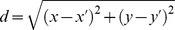. The kernel can be concave at the source with a fat–tailed distribution (*c*≤1), or convex and platykurtic (*c*>1), or can incorporate other important and well–known density functions as special cases, such as the square-root negative exponential (*c* = ½):
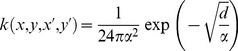
(3)the negative exponential (*c* = 1):
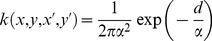
(4)and the Gaussian kernel (*c* = 2):
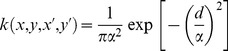
(5)Both the negative exponential and the Gaussian kernels are said to be ‘thin–tailed,’ meaning that the tails decline as fast as or faster than an exponential function. If the kernel is thin–tailed, the population advances at a constant velocity [19]–[21]. Ecologists interested in processes that operate at fine spatial scales, such as splash dispersal or the foraging behavior of small organisms, often use thin–tailed kernels. Kernels with fatter tails, such as the square-root negative exponential kernel (Eq. 3), lead to population expansion in ‘leaps and bounds’ ahead of the expanding wave, which means accelerating expansion [20], [22]–[24]. Ecologists interested in processes that operate at broad spatial scales, such as reforestation of habitat fragments and long–distance population spread commonly employ fat–tailed kernels [25].

**Figure 1:**
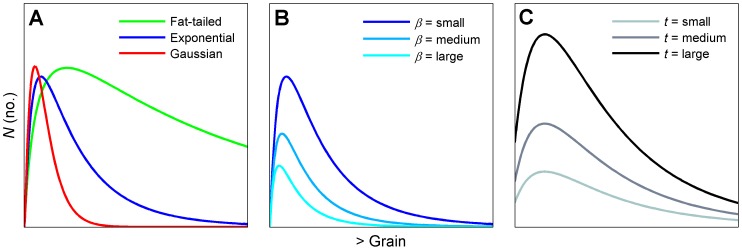
The dispersal scaling hypothesis (DSH). Increasing the spatial grain (patch size) of a habitat distribution relative to the gap–crossing abilities of a species produces a trade–off between the ‘benefits’ (larger patches = more dispersers) and ‘costs’ (dispersal distances become larger and harder to traverse) of increasing grain size. This results in a unimodal relationship between dispersal or spatiotemporal spread and spatial grain size. (**A**) Interaction of grain size and number of dispersing agents moving between a square donor patch and an adjacent, identical recipient patch using a simple continuous space model (Eq. 1), with three well known and fundamentally distinct classes of dispersal kernel (Eqs. 3–5). Kernels are parameterized so that they each have a mean dispersal distance of 1 m (i.e., *α* = 0.05, 0.5, and 1.13, respectively), *ρ* = 1 m^−2^, and grain size ranged from 1 to 100 m^2^. (**B**) Effect of parameter *β* as a multiplicative factor of the separation distance between donor and recipient patches in a fragmented habitat distribution, using the negative exponential kernel and grain sizes from **A**, and *β* = 1, 2, and 3. (**C**) Interaction of grain size and spatiotemporal development of a population within a recipient patch after multiple generations of spread from a donor patch, and subsequent population increase. A classic pairing of logistic differential equations for temporal and spatial population dynamics is used (Eq. 6), with: *N_0_* = 0.01 m^−2^; *ρ* = 1 m^−2^; *b* = 2 m^−1^; *r* = 0.025 day^−1^; *t* = 25, 50, and 75 days; and grain ranging from 0 to 25 m^2^.

To investigate the influence of spatial scale on the magnitude of dispersal, *N*, into the recipient patch, we successively substitute Eqs. 3–5 into Eq. 1, and increase donor and recipient patch dimensions, *L*, initially fixing *β* = 1 (i.e., patches are adjacent) (Fig. 1A). As grain size (*L* ^2^) increases, so does the quantity of dispersal agents, which facilitates dispersal from donor to recipient. Concomitantly, the distance from donor to recipient patch locations increases with grain, and dispersal is reduced. Thus, there is a trade–off between the ‘benefits’ (larger patches = more dispersers) and ‘costs’ (dispersal distances become larger and harder to traverse) of increasing grain size, due to the interplay between the characteristic scale of dispersal and the variable scale of resource patchiness. Consequently, the magnitude of dispersal, *N*, exhibits a scale-dependent optimum; i.e., theory predicts the existence of a unimodal (‘humpbacked’) relationship between the magnitude of dispersal and spatial scale of patchiness. Such unimodal functional relationships are pervasive in ecology; e.g., the unimodal relationship between habitat productivity and species richness has long been a central topic in ecology (for a review, see [26]). Note, however, that unimodal functions should not be confused with unimodal probability (frequency) distributions. A mode in the ordinary sense is a value that either appears most frequently in a sample or is the most likely value of a probability mass function. In contrast, the ‘mode’ of a unimodal function is not its most frequent value, as in a probability mass function; a unimodal function, *f* (*x*), is monotonically increasing for *x*≤*m* and monotonically decreasing for *x*≥*m*, where *m* is the value of the mode [26]. We see the same unimodal relationship when we vary *β* and introduce a gap between the patches (Fig. 1B). Indeed, this same relationship emerges under different parameterizations of the model than those used here in this example, provided the axes are scaled appropriately for the choice of *ρ* and *α*, and *β* is not so large as to completely prevent movement between the patches.

Thus far we have formalized the expected relationship between the magnitude of a single generation of dispersal and the scale of patchiness. We next extend this theoretical foundation to facilitate the linkage of both dispersal and population dynamics that give rise to spatiotemporal patterns of spread. Our hypothesis is that the same trade–off will continue to shape the relationship between *N* and grain size over multiple generations of dispersal and population growth. For instance, if we consider a pairing of logistic differential equations for temporal and spatial population dynamics (a classical pairing in theoretical epidemiology, e.g., [27]), the solution leads to the following equation:
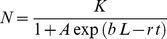
(6)where the carrying capacity *K* (no.) = *ρ L* ^2^ (the product of the patch population density parameter, *ρ*, and the recipient patch dimensions), *A* is the constant of integration = (*K*−*N_0_*)/*N_0_*, where *N_0_* is the theoretical *N* at the donor (0,0) at time = 0, *b* (length^−1^) is the rate of spatial spread, *L* is the distance from the center of the donor patch to the center of an adjacent recipient patch, *r* (time^−1^) is the growth rate of the population, and *t* is time. If we increase donor and recipient patch dimensions, we again find the same unimodal relationship between magnitude of spatiotemporal spread and scale at multiple values of *t* (Fig. 1C). Again, it should be noted that the same relationship emerges under different parameterizations of the model than that used in the example, provided the axes and length of time series are scaled appropriately for the choice of rate parameters.

### Spatially-explicit simulation of spread in complex landscapes

**Figure 2:**
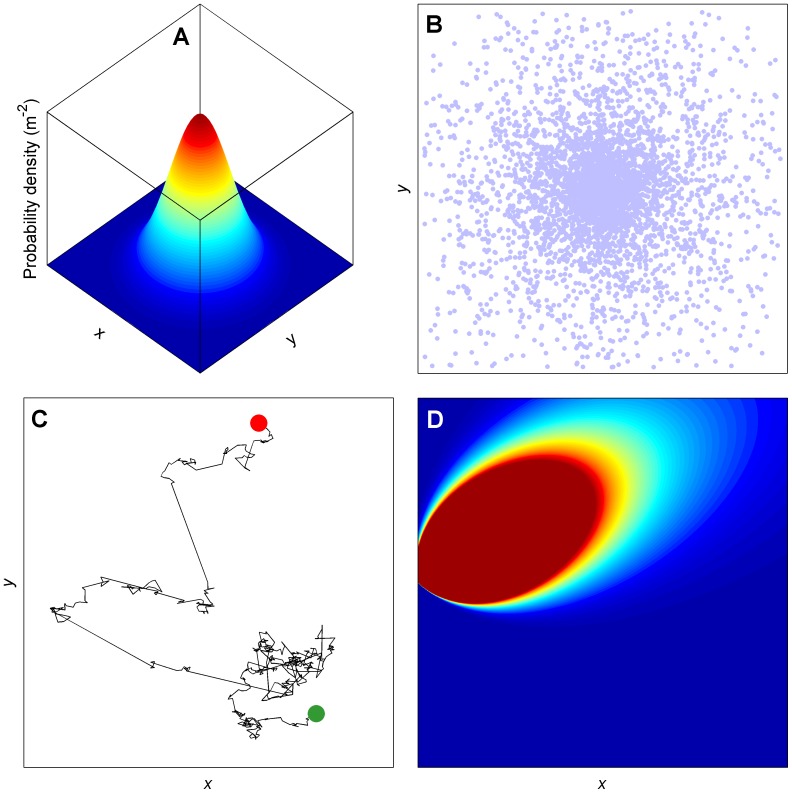
Example output from the various classes of dispersal model used in complex landscapes. (**A**) A symmetric, two–dimensional probability density function (dispersal kernel). Dispersal probability ranges from red (highest) to blue (lowest). The Gaussian kernel shown here is used for dispersal of a forest insect pest, *Dendroctonus ponderosae*. (**B**) Splash dispersal of a plant pathogenic bacteria, *Pseudomonas syringae*, using random draws from a negative exponential distribution for droplet distance and random draws from a uniform distribution for droplet angle. (**C**) A Lévy flight model for simulating the individual flight paths of a mosquito disease vector, *Culex erraticus*. Lévy flights are a special class of random walk that is punctuated by occasional long steps, and here we show the path of a mosquito flight beginning at the green marker and ending at the red marker. (**D**) A Gaussian plume model from the meteorological sciences used to simulate the dispersion of *Phytophthora infestans* (an oomycete plant pathogen) sporangia by wind and turbulence. Dispersal probability ranges from red (highest) to blue (lowest). The source of inoculum is situated in the center of the y–axis and the wind direction is 225 degrees.

To provide a more spatially-explicit evaluation of the generality of these theoretical predictions, we simulated the movement of a diverse range of economically important pests and diseases in complex landscapes. Collectively, these models encompassed both deterministic and stochastic methods of dispersal, individual– and population–level movements, as well as multiple generations of spatiotemporal spread (Fig. 2). We now consider a more elaborate description of space than the two–patch system evaluated mathematically above, given that patch size and gap size do not usually exhibit a positive correlation in the real world. Further, the distribution of distances between patches in a real landscape is unlikely to be uniform. Space was therefore simulated as a binary raster landscape (Cartesian grid) of host and non–host areas to create spatially heterogeneous landscapes in which various aspects of host pattern, such as host area, abundance, and degree of aggregation can be varied. We varied the grain size (cell dimensions) of landscapes to investigate the relationship between various attributes of landscape structure (which mimic different management strategies), scale, and infestation. In the following sections we first describe the species-specific models, the methods used for landscape generation, then the various pest and disease management scenarios we investigated.

### Species

#### Forest insect pest

Mountain pine beetle (*Dendroctonus ponderosae*) is a species of bark beetle native to the forests of western North America. It uses pine trees as hosts for brood production, a process of colonization that may cause the death of the host tree. In recent years, changes in forest demography and favorable weather conditions have opened the door to outbreak conditions, and the mountain pine beetle has become one of the most damaging of all forest–disturbance agents in the United States [28], [29]. Mountain pine beetles generally disperse over short distances (∼50 m) within the forest canopy [30] and the *D. ponderosae* model was based on Eq. 1 with a two–dimensional Gaussian dispersal kernel (Eq. 5) for beetle movement (Fig. 2A). This kernel has been used previously to simulate within-canopy dispersal of this and other species of bark beetle [29], [31]. The model was parameterized using empirically derived values from the literature for the density of beetles per host area, *ρ* = 59.4 m^−2^ [29], and the dispersal kernel spread parameter, *α* = 56 m, giving a mean dispersal distance of 50 m [30]. We considered a single generation of dispersal (a single redistribution event) where we summed the total number of beetles arriving at each host area from every other host area in the landscape; those redistributed to non-host areas were lost from the system. As this is a grid-based ecological model, this was accomplished efficiently with a two–dimensional, discrete convolution of the form [32]–[34]:
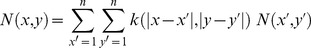
(7)where *k* (|*x−x*′|, |*y*−*y*′|) is the probability of moving from the centroid of donor cell (*x*′, *y*′) to the centroid of recipient cell (*x*, *y*) across two–dimensional space, and *N* (*x*′, *y*′) = *ρ*Δ*x*Δ*y* (the product of the patch population density parameter, *ρ*, and the cell dimensions). Convolutions were implemented via fast Fourier transforms, a technique that greatly enhances the speed and accuracy of the numerical solutions [12], [32], [35].

For each model run, we then averaged the number of beetles arriving across all host areas in the landscape:
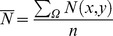
(8)The top summation quantifies the total number of beetles that dispersed to recipient patches in the landscape (a two-dimensional spatial domain, *Ω*), and the denominator, *n* (no.), is the total number of donor patches. In other words, 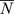 quantifies the average number of beetles that dispersed to another host patch on the landscape; it thus provides a global measure of the magnitude of infestation. As the grain size (cell dimensions) of landscapes is systematically increased, we obtained a distribution of 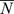 values that characterized the relationship between scale (grain size) and the magnitude of beetle infestation in landscapes. For within-canopy dispersal of mountain pine beetle, a wide range of grain sizes are relevant: from individual branches, to trees, up to large stands within a forest.

#### Bacterial plant pathogen

*Pseudomonas syringae* is a rod–shaped, Gram–negative bacterium that can infect a wide range of plant species; there are over 50 different pathovars [6]. The economic impact of *P. syringae* is increasing, with a resurgence of old diseases, including bacterial speck of tomato (pv. *tomato*; [36]) and the emergence of new infections of importance worldwide, such as bleeding canker of horse–chestnut (pv. *aesculi*; [37]). Several pathovars cause long–term problems in trees, often through the production of distortions and cankers (e.g. pathovars *savastanoi* and *morsprunorum*). Infections of annual crops are more sporadic, and outbreaks are often caused by sowing contaminated seed. Rain is an important mechanism for disseminating these micro–organisms from foliar surfaces, so we constructed a stochastic splash-dispersal model of *P. syringae* (Fig. 2B). Our model was based on a negative exponential distribution of dispersal distances, one of the most commonly used approaches in relevant studies [38]–[40]. All parameter values were obtained from a series of experiments that quantified the potential of (artificial) rain splash for removing and distributing *P. syringae* from oilseed rape foliage [41]. The initial bacterial population was set at 8×10^7^ bacterial cells m^−2^ foliage. We modelled a total of 7.5×10^4^ droplets m^−2^ foliage. All bacteria were washed off the leaves, and we assumed that each droplet contained an equal number of cells. Observed bacterial deposition gradients followed an exponential decline over distance; we therefore generated droplet dispersal distances, *ℓ* (m), by sampling a negative exponential distribution, using the inverse transform method [42], [43]:
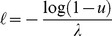
(9)where *u* is a real uniform random variable with values in (0,1), and *λ* is the rate parameter (set to 13.6 m^−1^, giving a mean dispersal distance of 7.35 cm [41]). The angle (*θ*, rad) of each droplet was a real uniform random variable with values in (0, 2*π*). Splash coordinates (*r*, *θ*) were converted to Cartesian coordinates (*x*, *y*), and we assigned each droplet a random starting location within the host cell, (*x_s_*, *y_s_*). Final droplet locations (*x_s_*+*x*, *y_s_*+*y*) were assigned to a grid cell in the landscape. This process was repeated, treating each host cell in the landscape as a source of bacteria, and as with the forest insect pest, we calculated the total number of bacterial cells arriving at each host cell from every other host cell in the landscape.

The global measure of infestation, 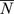, was calculated as before using Eq. 8. Through systematic increases in the grain size (cell dimensions) of landscapes, we obtained a distribution of 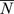 values that characterized the relationship between scale (grain size) and the magnitude of bacterial infestation in landscapes. For *P. syringae* dispersed by rain splash, a very narrow range of grain sizes are relevant: from individual leaves, to plants, up to rows or blocks of plants within a field.

#### Mosquito (disease vector)

The mosquito *Culex erraticus* is a vector for West Nile Virus (WNV), responsible for thousands of human cases and tens of thousands of avian and equine cases each year in the Americas [7]. It is also a vector for eastern equine encephalitis virus (EEEV), which is considered to be the most dangerous endemic arbovirus in the United States [44]; up to 70% of symptomatic cases in humans are fatal [45]. Besides the endemic and economic burdens to humans, frequent equine cases and sporadic mass game and wild bird die–offs are costly environmental consequences of WNV and EEEV transmission [46]–[48]. To simulate *C. erraticus* dispersal, we used a Lévy flight model that incorporated the perceptual range of the organism (Fig. 2C). A Lévy flight is a special class of random walks whose step lengths (*ℓ*) are best described by a power–law: *p* (*ℓ*)∼*ℓ* ^−*μ*^. Thus, there is no intrinsic scale to the step lengths, and very long steps can occur [49]. The exponent of the power law is named the Lévy index (1<*μ*≤3) and it controls the range of correlations in the movement, ranging from Brownian motion (*μ*>3) to straight–line paths (*μ*→1). Microorganisms, insects, birds, and mammals have been found to follow a Lévy distribution of flight lengths or times; moreover, it appears that *μ*≈2 is a common value for many species that exhibit Lévy flights [50]. For a comprehensive review of recent developments in the modeling of animal movement patterns as Lévy Flights see [51]. In the *C. erraticus* model, we set mosquito density *ρ* = 1 m^−2^ and simulated a separate Lévy flight of 50 steps for each individual mosquito, with an upper limit of 10 km as the maximum travel distance (all flight steps combined) [52]. For computational reasons, only the most central host cell in the landscape was initialized to contain a population of mosquitoes and we thus simulated individual flights to other host cells. Each Lévy flight started at a random location within the most central host cell, and we generated step lengths by sampling a power-law distribution using the inverse transform method [43], [53], [54]:
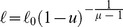
(10)where *u* is a real uniform random variable with values in (0,1), *ℓ_0_* is the minimum flight length (set to 20 m, the estimated perceptual range of a mosquito [52], [55]), and *μ* is the Lévy index (set to 2). The direction of each flight length (*θ*, rad) was a real uniform random variable with values in (0, 2*π*). Flight coordinates (*r*, *θ*) were converted to Cartesian coordinates (*x*, *y*), and the terminus of each step location was assigned to a grid cell in the landscape, which could be either a host or non-host cell.

The global measure of infestation, 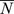, was calculated for each model run; however, as we were simulating dispersal events with multiple stops (each with the potential to spread a viral infection), *N* (*x*, *y*) in Eq. 8 was redefined as the total number of steps taken by all mosquitoes within that cell. Further, as there was only one donor cell of mosquitoes in each landscape, Eq. 8 could be reduced to the numerator only. This then gave the average number of potential infectious encounters per donor area in the landscape, as with the other species models. As the grain size (cell dimensions) of landscapes is systematically increased, we obtained a distribution of 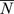 values that characterized the relationship between scale (grain size) and the magnitude of potential viral infestation in landscapes. For dispersing mosquitoes with a maximum flight range on the order of km, the relevant range of grain sizes vary from individual host organisms to successively larger groups and populations within a metapopulation.

#### Oomycete plant pathogen

*Phytophthora infestans* is an oomycete (formerly classified as a fungus) that is aggressive on many tuber and non–tuber bearing species of the genus *Solanum* [56]. It is the causal agent of potato late blight, which was responsible for the great Irish Potato Famine in the late 1840s, and is responsible today for multi–billion dollar losses annually in global tomato and potato production [8], [57]. *Phytophthora infestans* spreads through the production of massive amounts of sporangia, which are dispersed passively by wind and turbulence. This final species forms part of a detailed case study that uses weather data and a mechanistic simulation model of the potato late blight pathosystem [15]. We include this model here because it represents one of the few whole-pathosystem simulators where each component has been empirically parameterized and validated. It allows us to explore the generality of our findings using a detailed mechanistic model of the life cycle of both host and pathogen species, along with an atmospheric dispersion model from the meteorological sciences for long-distance dispersal of propagules. As the simulator is fully described elsewhere [14], [15], [58]–[61], we only summarize its salient characteristics here.

The simulator comprises two main components: a ‘within–field’ model and a ‘between–field’ model, both of which have been extensively tested against real data [15], [58], [60]. The within–field model simulates the growth of the host potato plant (foliage and tubers), the life cycle of the pathogen, host–pathogen interactions (as a function of potato variety and pathogen genotype), various fungicide management regimes, and the effects of the weather on all processes [14], [60], [61]. The between–field model is used to simulate the dispersal of inoculum among fields, and is comprised of models for the release of spores from sporangiophores, the escape of spores up through the canopy, long–distance transport of spores by wind and turbulence using a Gaussian plume model from the meteorological sciences (Fig. 2D), and mortality of spores during transportation due to exposure to UV radiation [15], [58], [59]. We used the simulator as described in Skelsey et al. [15]. Briefly, we simulated a spatial domain planted with a susceptible potato variety interspersed by non–host area. A single host cell was selected at random, and an epidemic initiated with 10 lesions m^−2^ ground area. Epidemics were allowed to progress for an entire growing season (1 May to 30 September 2012), driven by hourly weather from the Wageningen University “Haarweg” weather station (http://www.met.wau.nl/haarwegdata), 51°58′N, 5°38′E. We simulated standard fungicide applications in potato production: protectant fungicides were applied once per week in every host area, and eradicant sprays (that kill existing lesions) on an individual host area basis when disease severity breeched a certain threshold (≥1% disease severity).

The global measure of infestation, 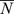, was calculated for each model run, with *N* (*x*, *y*) redefined as ‘disease incidence’ to account for the temporal dimension of this model; i.e., a ‘host encounter’ depended on the level of disease caused by the donor cell at the end of the growing season. Disease incidence is a common epidemiological measure for spatiotemporal epidemics; here, *N* (*x*, *y*) = 1 if the percentage of host tissue diseased ≥5%, or 0 otherwise. Thus, 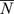 quantifies the average number of ‘host encounters’ per donor area in the landscape, as with the other species models. Through systematic increases in the grain size (cell dimensions) of landscapes, we obtained a distribution of 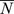 values that characterized the relationship between scale (grain size) and the magnitude of late blight infestation in landscapes. For spatiotemporal spread of late blight, where spores are dispersed over long distances by wind and turbulence, a very broad range of grain sizes are relevant: from individual leaves, to plants, up to fields, and large growing regions (containing many fields) within a country.

### Landscape generation

We used fractal geometry (the ‘inverse Fourier transform technique,’ [62]–[64]) to generate binary landscape patterns of host and non–host cells. We used fractal geometry because it creates natural-looking landscape patterns and facilitates tight control over the degree of aggregation of host cells via a single parameter known as the Hurst exponent, *H* [65], [66]. To fully evaluate interactions of host distribution, scale, and the magnitude of infestation, 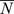, we generated a series of 50 landscapes of increasing grain size (the specific cell dimensions); in other words, each landscape pattern was generated at 50 different grain sizes. The specific range of grains sizes depended on the species being modeled, however. For example, splash dispersal of bacteria happens at very fine scales compared to long–distance dispersal of *P. infestans* sporangia by wind. We therefore defined the range of grain sizes to match the dispersal range of the organism of interest, which meant that a separate series of scaled landscapes was necessarily generated for each species. For the four species, the series of landscapes thus spanned the following range of grain sizes: 10^−2^ to 10^8^ m^2^ for *Dendroctonus ponderosae*, the mountain pine beetle; 10^−3^ to 10^2^ m^2^ for *Pseudomonas syringae*, the plant pathogenic bacterium; 10^1^ to 10^5^ m^2^ for the mosquito disease vector, *Culex erraticus*; and 10^0^ to 10^10^ m^2^ for *Phytophthora infestans*, the oomycete that causes potato late blight.

Landscapes were modeled as a 64×64-cell torus and were thus ‘wrapped,’ such that any mobile agent dispersing outside of the borders of the grid ‘reappeared’ on the opposite edge, equalizing immigration and emigration rates. This is a commonly used approach to approximate an infinite spatial extent (e.g., [67]–[70]). Thus, whilst the grain size of landscapes varied across a range of 50 values, spatial extent was effectively infinite. Moreover, the fractal technique we used generates ‘periodic’ landscape patterns that flow smoothly across borders when wrapped on a torus. This avoided individuals experiencing sharp discontinuities in host distributions when they crossed from one edge of the landscape and reappeared on the other, further reducing edge effects.

### Management scenarios

**Figure 3:**
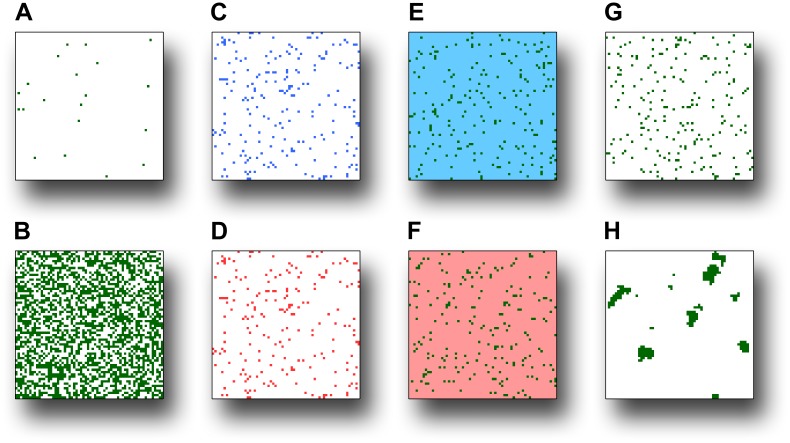
Example landscape patterns. We independently varied four aspects of host pattern between two extreme values in order to mimic different pest and disease management strategies: abundance of host areas, *h* (−), from (**A**) a low proportion in the landscape (*h* = 0.005) to (**B**) a high proportion (*h* = 0.5); quality of host areas, as determined by the pest or pathogen population density per square meter of host area, *ρ* (no. m^−2^), from (**C**) a low density (*ρ*/10), pictured as blue, to (**D**) high density (*ρ*×10), pictured as red; matrix resistance, i.e., the ability of organisms to move through the intervening matrix of non-host area, where dispersal distances between host areas were multiplied by a factor *R* (−), from (**E**) *R* = 0.1 to represent a highly permeable, low-resistance matrix, pictured as blue, to (**F**) *R* = 10 for a highly resistant matrix, pictured as red; and aggregation of host areas, *H* (Hurst exponent; −), from (**G**) a random distribution (*H* = −0.5) to (**H**) a highly aggregated distribution (*H* = 1). In each scenario, the remaining landscape parameters were fixed at baseline values of: *h* = 0.05, *ρ* as given for each species, *R* = 1, and *H* = −0.5.

We implemented the four species models under various landscape scenarios designed to mimic different pest and disease management strategies (Fig. 3). We independently varied four aspects of host pattern (landscape structure) between two extremes: (1) abundance of host areas, *h* (−), from a low proportion in the landscape (*h* = 0.005) to a high proportion (*h* = 0.5); (2) host quality, as determined by manipulation of the pest or pathogen population density per square meter of host area, *ρ* (no. m^−2^), from a low density (*ρ*/10) to a high density (*ρ*×10); (3) matrix resistance, i.e., the ability of organisms to move through the intervening matrix of non-host areas [71], which we simulated by adjusting dispersal distances between host areas by multiplying them either by a factor *R* = 0.1 to represent a highly permeable matrix (i.e., a matrix with low resistance to movement) or by a factor *R* = 10 for a highly resistant matrix; and (4) aggregation of host areas, *H* (−), which we adjusted to produce either a random distribution (*H* = −0.5) or a highly aggregated distribution (*H* = 1) of host areas (Fig. 3). Each of these landscape parameters were varied individually whilst the others remained fixed at intermediate values: *h* = 0.05, *ρ* as given for each species, *R* = 1, and *H* = −0.5.

It is helpful to give some examples as to how these landscape scenarios relate to specific pest and disease management strategies. Reduction of host abundance is akin to deployment of resistant plant varieties, eradication of hosts around detected infections, or animal vaccination programs. Reduction of host quality is commonly achieved through the use of chemicals, such as repellents, protectants, and biocides. Matrix resistance is often increased by the use of pheromone traps or trap plants (to lure pests), and in the case of airborne propagules (e.g., fungal spores), tall plants (such as maize) are often used as a barrier to shield valuable crops. Finally, host aggregation can be reduced through intercropping, mixed farming, or at a larger scale through restoration of biodiversity. Thus, these simple manipulations of host pattern encompass a wide range of pathosystem-specific management strategies.

A total of 500 simulations were performed for each scenario. In each model run, a different fractal map was generated. Values of 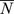 were averaged over the iterations. Relative standard errors of 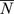 (standard error expressed as a percentage of the mean 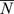) were calculated between iterations for each scenario, in order to assess the level to which the precision of simulation results were affected by variability in the random maps, and the number of repetitions performed.

## Results

**Figure 4:**
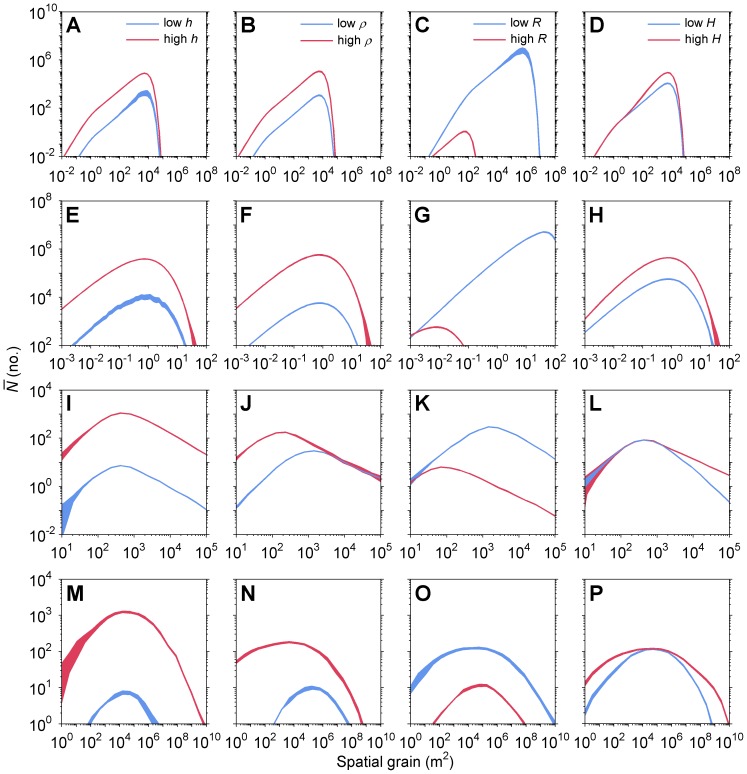
Interaction of grain size, host heterogeneity, and infestation. Magnitude of infestation, 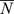 (no.), in landscapes where the spatial grain size (cell dimensions, m^2^) is systematically increased, under management scenarios that vary (across columns): host abundance, *h* (−); host quality, *ρ* (no. m^−2^); matrix resistance to movement, *R* (−); and aggregation of host areas, *H* (−). (**A**)–(**D**) Forest pest, the mountain pine beetle (*Dendroctonus ponderosae*); (**E**)–(**H**) plant pathogenic bacterium (*Pseudomonas syringae*); (**I**)–(**L**) mosquito disease vector (*Culex erraticus*); (**M**)–(**P**) spatiotemporal spread of the oomycete plant pathogen (*Phytophthora infestans*), the causal agent of potato late blight disease. Shaded regions indicate 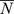
±1 SE. Fig. 2 provides examples of dispersal patterns, and Fig. 3 provides examples of landscape patterns.

Consistent with theoretical predictions ([4]; Fig. 1), there was a unimodal relationship between the magnitude of infestation, 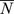, and spatial grain (the finest scale of patchiness in the host distribution) for all four species and under all management scenarios (Fig. 4). In all cases, there was a transition from increasing 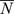 with increasing grain size up to some maximum level, at which point 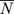 decreased with increasing grain size. While the specific maximum value of infestation clearly varied among species and management scenarios, the general unimodal response is qualitatively similar among species possessing a wide array of dispersal modes and ranges. This suggests, to us at least, that the DSH is a fairly robust phenomenon that holds up under a wide range of assumptions regarding dispersal, the scales at which it occurs, and the particulars of how space is modeled.

The various management techniques we simulated here had the potential to modify the shape of this relationship, but not the general unimodal form. For all four species, there were distinct domains of scale where management had the greatest potential to influence 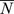. Changing host abundance (*h*) affected 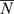 for the forest insect pest (Fig. 4A) and the bacterial plant pathogen (Fig. 4E) with a magnitude that decreased with increasing grain. This is because at coarser grain sizes limitations in the average dispersal distance or ‘gap–crossing’ abilities of the species contributed to a reduction in infestation. The number of neighboring areas had little impact on 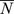 at the coarsest grain sizes, where dispersal became increasingly limited to immediate neighbors only (i.e., within the dispersal range of the species). Host abundance (*h*) had a comparatively greater impact on the mosquito (Fig. 4I) and the oomycete plant pathogen (Fig. 4M), and the size of the effect on 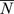 appeared constant across the range of grain sizes tested.

Varying the quality of host areas (*ρ*) had an almost identical effect to varying host abundance for the forest insect pest (Fig. 4B), and the bacterial plant pathogen (Fig. 4F). This suggests, for example, that a 10−fold change in the quality of host areas would be as effective in enhancing or disrupting spatial spread as a 10−fold increase or decrease in host abundance (% cover), respectively. Variation in host quality had a large effect on 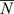 for mosquitoes at fine grain sizes and no effect after a grain size of approximately 10^3^ m^2^ (Fig. 4J). The ability of mosquitoes to reach distant host areas was already limited at coarse grain sizes (to ten steps or less), suggesting that the distances between host areas were more of a limiting factor in these landscapes. Similarly, variation in host quality had a diminishing effect on 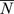 as grain size increased for the oomycete plant pathogen (Fig. 4N).

Changing the matrix resistance (*R*) to movement had the largest overall effect of the four management strategies tested. For all species but the oomycete plant pathogen, lowering *R* served to increase 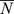 with a magnitude that increased markedly with grain size (Fig. 4C, 4G, 4K). Matrix resistance had such a large impact at coarse grain sizes because the distances between host areas that were already limiting movement became practically insurmountable (high *R*) or, conversely, now easily traversed (low *R*). For the oomycete plant pathogen, which has a large capacity for long–distance transport of inoculum via air currents, the effect of *R* on 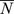 appeared constant across the range of grain sizes tested (Fig. 4O).

There were two distinct domains of scale in the effect of host aggregation on 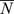 for the forest insect pest (Fig. 4D) and bacterial plant pathogen (Fig. 4H): an initial increase in the magnitude of the effect with grain size up to the mode, followed by a decrease or loss of any effect at coarser grain sizes. Host aggregation therefore had a greater impact over an ‘intermediate’ range of grain sizes (relative to the dispersal range of the organism), where the magnitude of 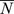 was ultimately maximized. This was not the case for mosquitoes, where host aggregation had no effect on 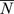 up to the grain size corresponding to the mode, but then there was a growing difference in the magnitude of the effect at coarser grain sizes (Fig. 4L). Interestingly, the effect of host aggregation on the forest insect pest and the bacterial plant pathogen was reversed in the case of the oomycete plant pathogen (Fig. 4P). There were three distinct domains of scale in the effect of host aggregation on 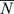: an initial decrease in the magnitude of the effect with increasing grain, an intermediate domain of no impact around the grain size corresponding to the mode of 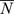, followed by an increase in magnitude once again at coarser grain sizes (Fig. 4P).

Relative standard error of 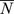 values, when averaged across all spatial scales and management scenarios, were low at 1% for the forest insect pest, 2% for the plant pathogenic bacterium, 7% for the mosquito disease vector, and 8% for the oomycete plant pathogen (Fig. 4). The level of replication was therefore deemed adequate for reducing noise in simulation outcomes to a level that did not obscure emergent patterns.

## Discussion

In this study we identified a potential for scale-dependent maxima in the magnitude of infestation for a variety of economically important pests and diseases under different landscape management scenarios. Our multi–model approach, encompassing deterministic and stochastic methods of dispersal, individual– and population–level movements, and multiple generations of spatiotemporal spread in complex landscapes, lends weight to the generality of the theoretical predictions of the DSH (Fig. 1). That the scale of environmental patchiness might interfere with pest and disease spread at extreme values of grain size should come as no surprise, however, as this is something that is already being exploited (or could be) in pest and disease management. For example, producers intentionally manipulate the grain of heterogeneity within their fields, such as by increasing crop diversity (e.g., using genotype mixtures or via intercropping), to slow disease spread for many fungal plant pathogens [72], [73]. At the other extreme, the grain of timber plantations within large production regions is also potentially limiting to the spread of fungal pathogens, as detached inoculum is often sensitive to ambient conditions and survival during transport is key to establishing disease over long distances [74], [75].

Our finding that various characteristics of spatial heterogeneity (the various landscape management scenarios) have the ability to shift the scale (grain size) at which the maximum level of infestation occurs, but not the occurrence of a maximum, is a previously unreported phenomenon that may have implications for the control of epidemics and pest outbreaks. Our model results suggest that the efficacy of efforts aimed at impeding the spread of pests or infectious agents are scale–dependent; what ‘works’ at one scale of host may have no effect at another. The DSH [4] predicts that spread among host areas will be maximized at an intermediate scale (grain size) of host heterogeneity (relative to the characteristic dispersal scale or gap–crossing abilities of the organism) (Fig. 1). However, our results clearly show that this is not necessarily the appropriate grain to target in managing disease spread (Fig. 4). For example, spread of potato late blight (the oomycete plant pathogen) could be limited to the site of epidemic initiation (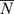
<1) in all four management scenarios through manipulation of heterogeneity at the finest and coarsest grains tested, and not the intermediate grain corresponding to the scale of maximum infestation (∼10^5^ m^2^) (Fig. 4M, 4N, 4O, 4P). In another example, mosquito dispersal among host areas was maximized at a grain size of approximately 10^3^ m^2^, but decreasing the quality of host areas was most effective at finer scales (Fig. 4J), and increasing matrix resistance and host aggregation was most effective at coarser scales (Fig. 4K, 4L). Ultimately, the appropriate scale to target in terms of managing disease spread depends not only on the species or pathosystem, but on what management technique or options are available or practical. Nonetheless, this occasional mismatch between the scale at which maximum infestation occurs and that for efficacious management raises some interesting questions: why the disparity, and what are the mechanisms behind it?

We can illustrate the complexity of these questions by considering the most striking example of variation in the efficacy of landscape management as a function of grain: the interaction of host aggregation (*H*) and the spread of potato late blight (oomycete plant pathogen; Fig. 4P). Host aggregation affected spread at extreme grain sizes, but had no effect over the middle range of scales where infestation was maximized. This surprising and somewhat counterintuitive result can be explained as follows. Increasing the degree of host aggregation shortens the average travel time between host areas, and spore travel time affects spread of potato late blight in a number of ways. Wind and turbulence serve to mix spore clouds with the surrounding air, leading to wider, deeper, and more dilute plumes with increased travel time. A shorter travel time between host areas at fine spatial grains therefore favors increased spore deposition, as spore clouds will be more shallow and concentrated. At intermediate grain sizes, the distances between host areas are larger and this process of plume expansion aids in the spread of disease over a wide area. A shorter travel time could therefore lead to a decrease in the number of areas infected at intermediate grain sizes. Detached sporangia are sensitive to ultraviolet radiation, and at very coarse grain sizes the distances between host areas could be severely limiting to spore survival during transportation [75]. Thus, a shorter travel time could lead to an increase in disease spread at very coarse grains.

The DSH may also have utility in the retrospective analysis of historical epidemic events. A recent study used historical data to suggest that a number of diseases caused by pathogens vectored by birds or spread by wind moved as accelerating wave fronts [76]. This type of disease spread indicates that host distribution (landscape pattern) had no effect on epidemic development. The results of our study also show a lack of an effect of host distribution, but only at certain scales. For instance, host aggregation had little effect on dispersal at fine grain sizes for the mosquito disease vector (Fig. 4L), and little effect at extreme grain sizes for the forest insect pest (Fig. 4D), and the bacterial plant pathogen (Fig. 4H), and thus accelerating waves of disease could be expected in these domains. For potato late blight, host aggregation had no effect on spread of disease at intermediate grain sizes where disease spread was maximized (Fig. 4P), and accelerating waves of disease could be expected over this range. Notably, this range includes the grain sizes at which potato was most commonly grown during the ‘European Potato Murrain’ of the 1840's; from the numerous marginal plots of the farm laborer (e.g., 0.1 ha ‘conacres’; [77]), to the larger fields where harvest was destined for industrial processing [78]. Indeed, maps of epidemic fronts constructed from archival publications show accelerating waves of disease for potato late blight during the Potato Murrain [76], [79].

A number of constraints on our findings should be noted. Although the suite of models used to test the generality of the DSH did include an individual-based model of animal movement that incorporated the effects of a perceptual range on animal movement (the mosquito disease vector), we did not address adaptive changes in movement in response to grain. For example, foraging movements among small host areas may differ from long–distance movements between large host areas, as dispersal bears a cost and behavioral responses aimed at reducing that cost may occur. In addition, a number of factors may influence an organism's willingness to approach, move away from, or cross the edge of a patch, such as the quality of resources within the host area or the type of habitat surrounding the host area (i.e., the patch context). An important next step is thus to test our theoretical predictions using more mechanistic models of animal vector movement. These should include models for dispersal in the marine environment, which is also threatened worldwide by the spread of pathogenic and invasive organisms [80], [81]. It would also be beneficial to compare theoretical predictions with empirical data, but we cannot yet fully confront the DSH with data given the absence of datasets on dispersal or spatiotemporal spread that span such a large range of scales. Nevertheless, we do provide a modeling framework to generate predictions that can inform and streamline future experimental or empirical validation studies. For instance, a distribution of 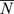 (Fig. 4) can be used to identify distinct spatial domains where three sets of measurements could be obtained to test the DSH, to confirm if there is a marked shift from an increase to a decrease in dispersal or spatiotemporal spread once a threshold in grain size of heterogeneity is crossed. Such information would in turn be valuable for monitoring and sampling spatial spread, as opportunities for species movement will be highest at the scale(s) where dispersal success is maximized. If management intervention is required, then this framework may be used to identify the grain sizes and attributes of host heterogeneity at which intervention may be efficacious, which our results suggest are likely to be specific to the species and management technique in question.
